# Role of transient receptor potential channels on pathogenesis and treatment of psoriasis

**DOI:** 10.1007/s10787-025-02095-0

**Published:** 2026-02-14

**Authors:** Ana Merian da Silva, Romina Nassini, Francesco De Logu, Juliano Ferreira

**Affiliations:** 1 Graduate Program in Pharmacology, Federal University of Santa Catarina (UFSC), Florianópolis, Santa Catarina 88037-000 Brazil; 2 Department of Health Sciences, Clinical Pharmacology and Oncology Section, University of Florence, 50139 Florence, Italy; 3 Department of Pharmacology, Biological Science Center, Block D, Federal University of Santa Catarina (UFSC), Trindade, Florianópolis, SC 88040-900 Brazil

**Keywords:** Dermatitis, TRP channels, Drug target, Adverse effects

## Abstract

Psoriasis is a chronic inflammatory skin disease characterized primarily by hyperproliferation of keratinocytes, infiltration and activation of immune cells, including T lymphocytes and macrophages, as well as increased innervation by sensory neurons. Although several therapeutic options are available, the management of psoriasis remains unsatisfactory, with adverse effects and unmet clinical needs. In this context, channels from the Transient Receptor Potential (TRP) family, which are non-selective cation channels involved in various pathologies, have been identified as potential therapeutic targets for treating psoriasis. Growing evidence suggests the involvement of multiple TRP subtypes in the pathogenesis of psoriasis, including altered expression of vanilloid subtypes, such as TRPV1, TRPV3, TRPV4, TRPV6, the canonical TRPC6, and melastatin TRPM8 in patients. These channels are involved in processes such as keratinocyte differentiation and proliferation, immune cell activation (e.g., T cells), and sensory neuron stimulation. Although there are still few studies on the role of TRPs in the therapies currently used for psoriasis, there is evidence of the activation of TRPV1 and the TRPA1 subtypes in the adverse effects of topical pharmacotherapy and phototherapy. On the other hand, TRPV1 desensitization (usually produced by repeated treatment with the TRPV1 agonist capsaicin) can reduce the severity of psoriasis and pruritus. Thus, the pharmacological modulation of TRP channels represents a promising strategy for developing novel, efficacious, and safer therapies to treat patients with psoriasis. This review aimed to provide a comprehensive overview of the involvement of TRP channels in the pathogenesis and therapeutic approaches to psoriasis.

## Introduction

Psoriasis is a chronic inflammatory skin disease characterized by thickened and scaly plaques. Psoriasis affects approximately 3% of the world’s population and may present with both cutaneous and systemic manifestations, as well as substantial adverse effects on patient quality of life (Elmets et al. 2021). Psoriasis also has different phenotypic subsets, defined by the location of clinical characteristics, such as nail psoriasis, scalp psoriasis, and palmoplantar psoriasis (Rendon and Schäkel 2019). Additionally, various morphologies exist, including guttate psoriasis, pustular psoriasis, and chronic plaque psoriasis (Griffiths et al. 2021). Studies have shown that these individual variables, extent, and severity may be predictors of inflammation evolution, with approximately one-third of patients with psoriasis transitioning to psoriatic arthritis (PAs) (Scher et al. 2019).

The pathogenesis of psoriasis is complex, and the exact mechanism remains elusive (Armstrong and Read 2020). Psoriasis is believed to result from a combination of genetic, epigenetic, and environmental factors. Pathophysiology is characterized by abnormal keratinocyte proliferation and immune cell infiltration in the dermis and epidermis, involving both the innate and adaptive immune systems, with dendritic cells (DCs) and T cells playing essential roles (Greb et al. 2016; Sieminska et al. 2024). The interplay of immune cells and cytokines is a critical factor in the pathogenesis of psoriasis (Polese et al. 2020). T helper 17 (Th17) cells is believed to be particularly important in psoriasis due to its proinflammatory effects and involvement in an integrated inflammatory loop with dendritic cells and keratinocytes, contributing to an overproduction of antimicrobial peptides, inflammatory cytokines, e.g.: tumor necrosis factor alpha (TNF-α), interleukin (IL)-17, IL-22 and IL-23, and chemokines (e.g. monocyte chemoattractant Protein-1 (MCP-1/CCL2)) that leads to the amplification of the immune response. In addition, other pathways and signaling molecules are involved, including macrophages, neutrophils, and gamma-delta T (γδ T) cells, as well as their related cytokines (for a comprehensive review of signaling pathways involving extracellular cytokines and intracellular transmission, see Guo et al. 2023; Sieminska et al. 2024).

Sensory symptoms associated with psoriasis, such as itch and pain, can negatively impact patients’ well-being (Kaaz et al. 2019). Itch is a significant indicator of psoriasis and has been reported in 60–90% of patients. Studies from the last decade have elucidated the pathogenesis of psoriasis patients’ sensory-related symptoms (especially itch) (for a review, see: Kaczmarska et al. 2023). Sensory nerve fibers more densely innervate psoriasis skin compared to non-psoriasis skin (Kubanov et al. 2015), with increased expression of Protein Gene Product 9.5 (PGP9.5), a commonly used pan-neuronal marker, and a positive correlation with the intensity of pruritus in patients with psoriasis (Kupczyk et al. 2018). The nervous system is involved not only in sensory symptoms but also in the disease itself. According to studies, patients with psoriasis who have nerve damage have shown unilateral local improvement and even complete remission of the condition at the affected site (Zhu et al. 2016). The mechanism by which nerve damage can lead to remission of psoriasis involves a decrease in neuropeptides produced and secreted by peripheral neurons, such as calcitonin gene-related peptide (CGRP), which influence the infiltration of immune cells and epidermal hyperplasia (Ostrowski et al. 2011; Yin et al. 2022). Finally, psoriatic keratinocytes increase the production of neurotrophic factors, including Nerve Growth Factor (NGF), which positively stimulates sensory neurons and upregulates neuropeptides responsible for maintaining the inflammatory cascade and inducing the neurogenic pruritus of psoriasis (Raychaudhuri et al. 2008; Shang et al. 2023). Given this, this review aimed to provide a comprehensive overview of the involvement of TRP channels in the pathogenesis and therapeutic approaches to psoriasis.

## Role of TRP channels in psoriasis pathogenesis

**Fig. 1:**
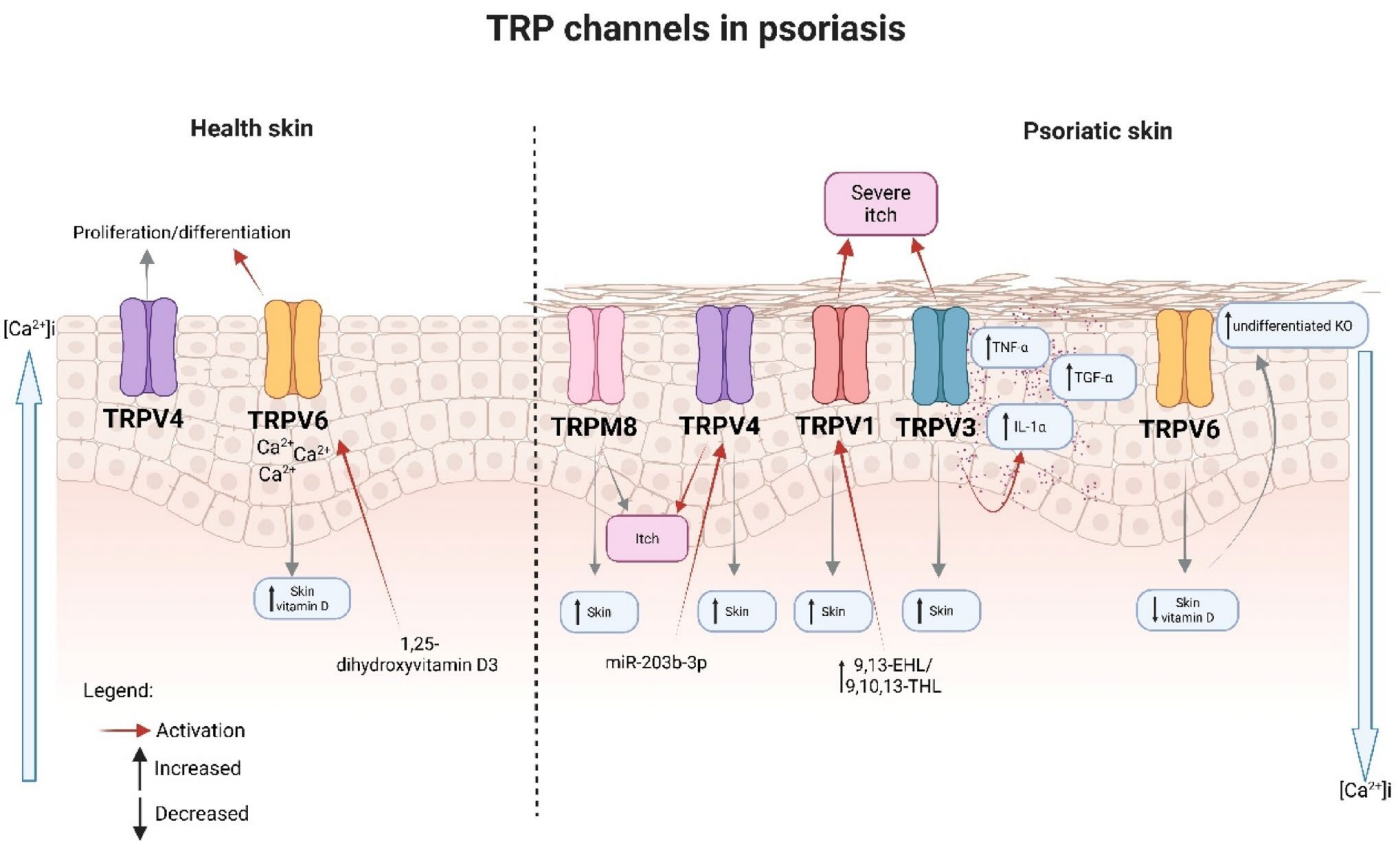
Role of TRP Channels in Psoriasis Pathogenesis. Several lines of evidence demonstrate abnormal function of TRP channels in psoriasis, due to either excessive or deficient activity. Reduced expression of TRPV6 (a channel regulated by 1,25-dihydroxyvitamin D3) disrupts the calcium gradient necessary for proper epidermal differentiation and favors the proliferation of undifferentiated keratinocytes. Other channels are directly associated with itch intensity, such as TRPV1, which shows increased levels in damaged and pruritic skin, accompanied by higher levels of its lipid activators (9,13-EHL and 9,10,13-THL). TRPV3 also shows elevated expression, and its activation in keratinocytes induces cell proliferation and promotes the release of TNF-α, TGF-α, and IL-1α, along with EGFR activation, contributing to keratinocyte hyperproliferation. Psoriatic keratinocytes also exhibit increased expression of TRPV4, which can be indirectly activated by microRNAs overexpressed in lesions, particularly miR-203b-3p, known to induce calcium influx in sensory neurons and scratching behavior through PKC-dependent TRPV4 phosphorylation. Another overexpressed channel in pruritic lesioned skin is TRPM8, whose expression correlates directly with the intensity of itching

Mammalian Transient Receptor Potential (TRP) channels have six subfamilies, including TRPA (Ankyrin), TRPC (Canonical), TRPM (Melastatin), TRPML (Mucolipin), TRPP (Polycystin), and TRPV (Vanilloid), with six transmembrane domains, with the N- and C-terminal regions located inside the cell and assembled as homo- or hetero-tetramers to form non-selective cation-permeable pores (Blair et al. 2023). TRP channel activation in response to chemical and physical stimuli typically promotes cation influx (especially [Ca^2+^]i) and cell excitability in various cellular processes (Zhang et al. 2023). TRP channels not only act as ‘polymodal cellular sensors’ on sensory neurons but are also functionally expressed by many non-neuronal cell types. Especially in the skin, where they appear to be critically involved in regulating various cutaneous functions both under physiological and pathophysiological conditions (Koivisto et al. 2022). A growing body of evidence implicates abnormal TRP channel function, resulting from excessive or deficient channel activity, in various pathological skin conditions (Caterina and Pang 2016; Zhang et al. 2023; Zhou et al. 2018, 2019). Thus, TRP channels are pharmacological targets in many skin diseases, including skin cancers, chronic itch, dermatitis, vitiligo, alopecia, and psoriasis (Fig. 1). Psoriatic skin lesions are characterized by thickening of the epidermis because of increased keratinocyte proliferation accompanied by reduced keratinocyte differentiation (Griffiths et al. 2021). One important factor that regulates cellular differentiation is Ca^2+^ (Leuner et al. 2011). In normal epidermis, the Ca^2+^ concentration is highest in the granular layer, while that in the basal layer is the lowest, forming a gradient that is disturbed in psoriatic plaques, which favors impaired keratinocyte proliferation (Cubillos and Norgauer 2016).

Several lines of evidence suggest a role for TRP channels in the pathogenesis of psoriasis, including altered expression of TRP channels in patients with psoriasis (Table 1). The first clinical evidence indicating a possible role of TRP channels in psoriasis was related to TRPV6, a protein regulated by 1,25-dihydroxyvitamin D3. When compared with healthy subjects, TRPV6 mRNA and protein were low in the lesional skin of psoriasis patients with or without joint involvement (Cubillos and Norgauer 2016). Notably, TRPV6 is a key element in the differentiation of human keratinocytes in response to Ca^2+^ and 1,25-dihydroxyvitamin D3 (Lehen’kyi et al. 2007), a highly efficacious antipsoriatic drug (see therapy section below). Moreover, patients with psoriasis typically have lower vitamin D levels than healthy subjects, and a negative correlation exists between serum vitamin D levels and disease duration and severity (Mohta and Nyati 2022).

**Table 1:** Possible role of TRP channels in psoriasis

TRP channel	Expression in patients	Activators in the patients	Animal model of psoriasis	Patient with psoriasis keratinocytes	Healthy keratinocytes
TRPV6	↓ Skin	↓ Vitamin D	N.D	N.D	↑ differentiation induced by 1,25-dihydroxyvitamin D3
TRPV1	↑ Skin & PBMC	↑ 9,13-EHL & 9,10,13-THL	Knockout, antagonism & defunctionalization reduced dermatitis	N.D	↑ differentiation induced by acid
TRPV3	↑ Skin ↓ PBMC	N.D	N.D	↑ release of IL-1-α	↑ release of TNF-α and TGF-α
TRPV4	↑ Skin ↓ PBMC	↑lysophosphatidylcholine & miR-203b-3p	Knockout and antagonism reduced dermatitis	N.D	↑ differentiation and proliferation
TRPA1	↑/ ↔ Skin ↔ PBMC	N.D	Knockout reduced or increased dermatitis incidence	N.D	↑ differentiation and inflammation-related genes
TRPM8	↑ Skin	N.D	Agonism reduces dermatitis and itch	N.D	Regulate cold-induced differentiation
TRPC1	↔ PBMC	N.D	N.D	Reduced expression	Mediated differentiation induced by calcium
TRPC4	N.D	N.D	Knockdown reduced itch	Reduced expression	N.D
TRPC6	↑ PBMC	↑ diacylglycerol	N.D	Mediated calcium entry defect	Calcium-mediated differentiation

N.D., Nondetermined; ↑, Increased; ↓, Decreased; ↔ , Not changed

TRPV1 is activated at high temperatures (~ 43 °C) as well as by some exogenous (e.g., the pungent ingredient of chili peppers, capsaicin) and endogenous substances (protons and some polyunsaturated fatty acids), and there are several lines of clinical and non-clinical evidence indicating its role in psoriasis (Arora et al. 2021). Stimulation of TRPV1 by protons induced differentiation of cultured human keratinocytes (Denda et al. 2010). Compared with healthy skin, itchy lesional skin of psoriasis patients presented increased expression of TRPV1 mRNA and protein (Nattkemper et al. 2018). Similarly, the linoleic acid-derived mediators 9,10-epoxy-13-hydroxy-octadecenoate (9,13-EHL) and 9,10,13-trihydroxy-octadecenoate (9,10,13-THL), which are endogenous activators of TRPV1, were found to be more concentrated in lesions of psoriasis patients compared with those of healthy individuals (Wheeler et al. 2022). More clinical evidence for a role of TRPV1 in psoriasis includes the antipsoriatic effect of topical capsaicin (a TRPV1 agonist) in patients (see therapy section below) and the reduction of psoriasis severity and itch by an NGF receptor TrkA inhibitor in patients, through the TRPV1 pathway (Roblin et al. 2015).

Compared to wild-type mice, mice with TRPV1 gene deletion have a reduction in skin inflammation and barrier defects induced by topical imiquimod, a model of psoriasiform dermatitis (Zhou et al. 2018). Additionally, TRPV1 knockout or antagonism reduced cutaneous discomfort associated with pruritus in the same murine model of psoriasis (Kodji et al. 2019). The apparent discrepancy between clinical (agonism presenting antipsoriatic effect) and non-clinical (knockout or antagonism presenting antipsoriatic effect) may be explained by capsaicin-induced short-lasting stimulation of TRPV1, followed by a long-lasting defunctionalization of TRPV1-expressing cells (predominantly sensory neurons, named TRPV1-positive neurons) (Arora et al. 2021). The defunctionalization of TRPV1-positive neurons reduced not only imiquimod-induced skin inflammation in mice but also impaired the ability of dermal dendritic cells to produce IL-23-dependent inflammatory cytokines by dermal γδT cells and the subsequent recruitment of inflammatory cells to the skin (Riol-Blanco et al. 2014). Reinforcing these findings, the endogenous lipid resolvin D3 also reduced imiquimod-induced skin inflammation and itch in mice by inhibiting TRPV1 activation and CGRP release from sensory neurons (Lee et al. 2020).

TRPV3 is activated at innocuous temperatures (~ 33 °C) and by certain endogenous substances, such as farnesyl pyrophosphate (FPP), an intermediate metabolite in the mevalonate pathway (Bang et al. 2010). TRPV3 mRNA levels were higher in lesional skin than in non-lesional skin or in healthy subjects (Larkin et al. 2021). Moreover, TRPV3 activation in human keratinocytes leads to proliferation. It releases TNF-α, a relevant target for antipsoriatic drugs (see therapy section below), as well as transforming growth factor alpha (TGF-α), a mitogen implicated in the hyperproliferation of keratinocytes in psoriasis patients (Turbitt et al. 1990; Szöllősi et al. 2018; Wang et al. 2021). Stimulation of TRPV3 in keratinocytes from psoriasis patients induces the release of interleukin-1α (IL-1α) and activates the epidermal growth factor receptor (EGFR) (Scott et al. 2016). TRPV3 forms a signaling complex with EGFR in keratinocytes, and activation of the epidermal growth factor receptor signaling pathway occurs in lesional skin from psoriasis patients. Activation of TRPV3 led to EGFR signaling pathway activation in human primary keratinocytes, an effect inhibited by TRPV3 antagonists. TRPV3 mRNA is even more increased in pruritic, compared to nonpruritic, lesional skin of psoriasis patients as well, and its expression was correlated with itch intensity (Nattkemper et al. 2018, 2023). Indicating it as a target to treat psoriasis, the use of TRPV3 in developing a drug for the treatment of psoriasis is protected by an international patent (Wang et al. 2021; World Intellectual Property Organization 2019).

TRPV4 is a detector not only of thermal, mechanical, and environmental cues, but also of endogenous substances (e.g., lysophospholipids) (Benítez-Angeles et al. 2023). Notably, lysophosphatidylcholine levels were significantly increased in both the plasma and skin lesions of psoriatic patients, facilitating the pathogenesis of psoriasis by activating keratinocytes and T cells (Liu et al. 2023). TRPV4 knockdown or antagonism reduced the proliferation of human keratinocytes (Amalia et al. 2023). Compared to healthy skin, keratinocytes in human psoriasis skin had high TRPV4 expression. Moreover, Trpv4 knockout or antagonism in mice resulted in reduced dermatitis, T cell and macrophage infiltration, and decreased expression of IL-17, IL-23, and CGRP compared to wild-type mice in the imiquimod-induced model (Amalia et al. 2023). Moreover, experiments using an animal model of psoriasis demonstrated that IL-17 receptor activation upregulated the channel TRPV4 in sensory neurons, inducing a psoriasis-like itch (Zhang et al. 2025). Finally, TRPV4 may be indirectly activated in psoriasis skin, as human psoriatic lesions have been shown to exhibit increased expression of several microRNAs, including miR-203b-3p. This microRNA induces a calcium ion response in sensory neurons and scratching behavior in mice through protein kinase C-dependent phosphorylation of TRPV4 (De Logu et al. 2023). Additionally, evidence suggests that TRPV4 antagonists have not been clinically tested in patients with psoriasis, while they are being developed to treat cardiovascular diseases (Fan et al. 2024).

TRPA1 is also a polymodal cellular receptor to several endogenous and exogenous stimuli. It has been demonstrated that TRPA1 activation in human keratinocytes increases the expression of genes that regulate cell proliferation and inflammation (Atoyan et al. 2009). The role of TRPA1 in psoriasis has been primarily explored in a mouse model of psoriasiform dermatitis induced by imiquimod, with divergent results depending on the study. Kemény et al. (2018) demonstrated that TRPA1 knockout or antagonism worsened skin inflammation and itchy behavior in mice, suggesting a protective role for TRPA1 in psoriasis. On the other hand, Zhou et al. (2019) reported that TRPA1 gene deletion reduced the late-stage skin inflammation, neutrophil and T cell recruitment, and the expression of IL-17, IL-22, and IL-23, indicating a hampering effect of TRPA1 in psoriasis. Divergent results were also found in terms of TRPA1 expression. While Zhou et al. (2019) found an increase in TRPA1 expression in the lesional skin of psoriasis patients, Nattkemper et al. (2018) were unable to find any differences in TRPA1 expression between the lesional skin of psoriasis patients and the skin of healthy subjects. Thus, the role of TRPA1 in psoriasis pathogenesis remains uncertain.

TRPM8 is activated at cold temperatures and by some exogenous (e.g., menthol and thymol) and endogenous substances (such as phosphatidylinositol 4,5-bisphosphate) (Liu and Qin 2005). TRPM8 mRNA has increased in pruritic, compared to nonpruritic, lesional skin of psoriasis patients, and its expression is correlated with itch intensity (Nattkemper et al. 2018, 2023). Furthermore, thymol treatment reduced skin dermatitis, itchy behavior, neutrophil infiltration, dendritic cell infiltration, and Th17 cell infiltration, as well as decreased TNF-α, IL-17, IL-22, and IL-23 in the skin of imiquimod-treated mice, via activation of the TRPM8 channel (Wang et al. 2020). Notably, an epidermal isoform of TRPM8 (eTRPM8) has been identified, which regulates cold-dependent keratinocyte proliferation and differentiation (Bidaux et al. 2015). Unfortunately, clinically available TRPM8 modulators have not been tested in patients with psoriasis.

TRPM4 is involved in diverse physiological processes and implicated in several human hereditary diseases. Notably, gain-of-function mutations of TRPM4 predispose mice to psoriasiform dermatitis, characterized by an increased accumulation of γδ T cells, elevated IL-17 expression, enhanced migration of dendritic cells, and increased keratinocyte proliferation (Yamada et al. 2022). Recently, the first case of an elderly patient with generalized pustular psoriasis and a TRPM4 mutation was presented, suggesting a potential association between TRPM4 and pustular psoriasis (Hsieh et al. 2015).

Some evidence also demonstrated differential TRP channel expression in peripheral blood mononuclear cells of psoriasis patients. A decrease in TRPM4, TRPM7, TRPV3, TRPV4, or TRPC6 mRNA expression and an increase in TRPM2 or TRPV1 mRNA expression levels were observed in the peripheral blood mononuclear cells of psoriasis patients, compared to healthy subjects (Özcan et al. 2021). There was no significant difference in TRPA1, TRPV2, TRPM1, TRPM5, TRPC1, TRPC2, TRPC3, and TRPC5 genes between the patient and control groups. Moreover, a negative correlation was found between TRPV3 and TRPC6 mRNA expression and the Psoriasis Area and Severity Index (PASI) value (Özcan et al. 2021). These results have some limitations, including the need for additional research on protein expression and a comparison with skin samples from patients with psoriasis.

Finally, there is also evidence from keratinocyte cultures of healthy and psoriasis patients, indicating the role of members of the TRPC channels in psoriasis. TRPC channels have frequently been proposed to act as store-operated channels (SOCs), which are activated by the depletion of intracellular calcium stores (Blair et al. 2023). Initially, mRNAs for TRPC1, TRPC5, TRPC6, and TRPC7 were detected in undifferentiated human keratinocytes (Cai et al. 2005). Their mRNA levels initially increased, then decreased during calcium-induced differentiation. TRPC1 alone may not form a functional ion channel, but it does form heteromers with TRPC4 and TRPC5 (Blair et al. 2023). Keratinocytes with disrupted TRPC1 or TRPC6 expression exhibited decreased calcium-induced differentiation (Cai et al. 2006; Müller et al. 2008). Furthermore, keratinocytes from psoriasis patients presented substantial defects in Ca^2+^ influx in response to high extracellular Ca^2+^, associated with a downregulation of TRPC1, TRPC3, TRPC4, TRPC5, TRPC6, and TRPC7. Moreover, TRPC6 channel activation partially improved the Ca^2+^ entry defect (Leuner et al. 2011). Notably, diacylglycerol is an endogenous activator of TRPC6, and its level is elevated in psoriatic skin (Fisher et al. 1990; Hofmann et al. 1999). Finally, TRPC4 was expressed in sensory neurons, and its knockdown reduced itchy behaviours induced by imiquimod in mice (Lee et al. 2020).

Besides the presented evidence of TRP channels as targets for treating psoriasis, their modulators need to be tested in patients to confirm their therapeutic potential.

## Psoriasis therapy

Although numerous treatments are available, the management of psoriasis is considered inadequate, presenting several drawbacks, including adverse effects and unmet needs that persist (Lee and Kim 2023). The treatment of psoriasis depends on the extent and severity of the disease, as well as the involvement of specific areas and the presence of psoriatic arthritis. Psoriasis may be treated with topical or systemic drugs and phototherapy. Most psoriasis patients present with mild to moderate disease, which in many cases can be successfully treated with topical agents, typically used in patients with mild to moderate psoriasis, involving less than 10% of the body surface area (BSA) (Elmets et al. 2021; Goddard et al. 2025). Topical corticosteroids are recommended for treating plaque psoriasis that does not involve intertriginous areas. Steroid-sparing agents, such as vitamin D analogs, tazarotene, and calcineurin inhibitors, can be used alone or in combination with steroids to treat psoriasis with a lower risk of steroid-induced adverse effects. The alternate use of steroids and steroid-sparing agents plays a critical role in the chronic management of psoriasis. Other topical agents, such as dithranol and capsaicin, can also be used alone or in combination with topical steroids for treating psoriasis. Topical treatments can be combined with biological and other systemic agents to increase the efficacy of therapy.

Patients are candidates for systemic non-biological pharmacotherapy when lesions cover more than 10% of the BSA and/or when the disease is present in specific regions (for a comprehensive review of non-biological therapy, see Menter et al. 2020). Methotrexate and apremilast are recommended for the treatment of moderate to severe psoriasis in adults. Methotrexate is less effective than biological TNF inhibitors for cutaneous psoriasis. Cyclosporine is recommended for patients with severe, recalcitrant psoriasis. Acitretin is recommended as a monotherapy or as a combination therapy with phototherapy, and it should not be used in patients who are pregnant or intend to become pregnant or are nursing. Other non-biological drugs used to treat psoriasis include: fumaric acid esters, tofacitinib, mycophenolate mofetil, azathioprine, leflunomide, and tacrolimus. Patients with psoriasis are candidates for systemic biological therapy when they present with moderate to severe disease and/or when the disease is present in specific areas, such as joints (for a comprehensive review of systemic biological therapy, see Menter et al. 2019).

Patients with psoriasis who do not have any photosensitivity disorders or a risk of skin cancer may be treated with phototherapy (Elmets et al. 2019). Phototherapy modalities may be used as monotherapy or in combination with other psoriasis therapies to treat moderate to severe psoriasis in adults. These modalities include narrowband ultraviolet B (NB-UVB), broadband ultraviolet B (BB-UVB), or targeted band UVB, UVA with psoralens (PUVA), and photodynamic therapy (utilizing photosensitizing chemicals, such as 5-aminolevulinic acid).

**Fig. 2:**
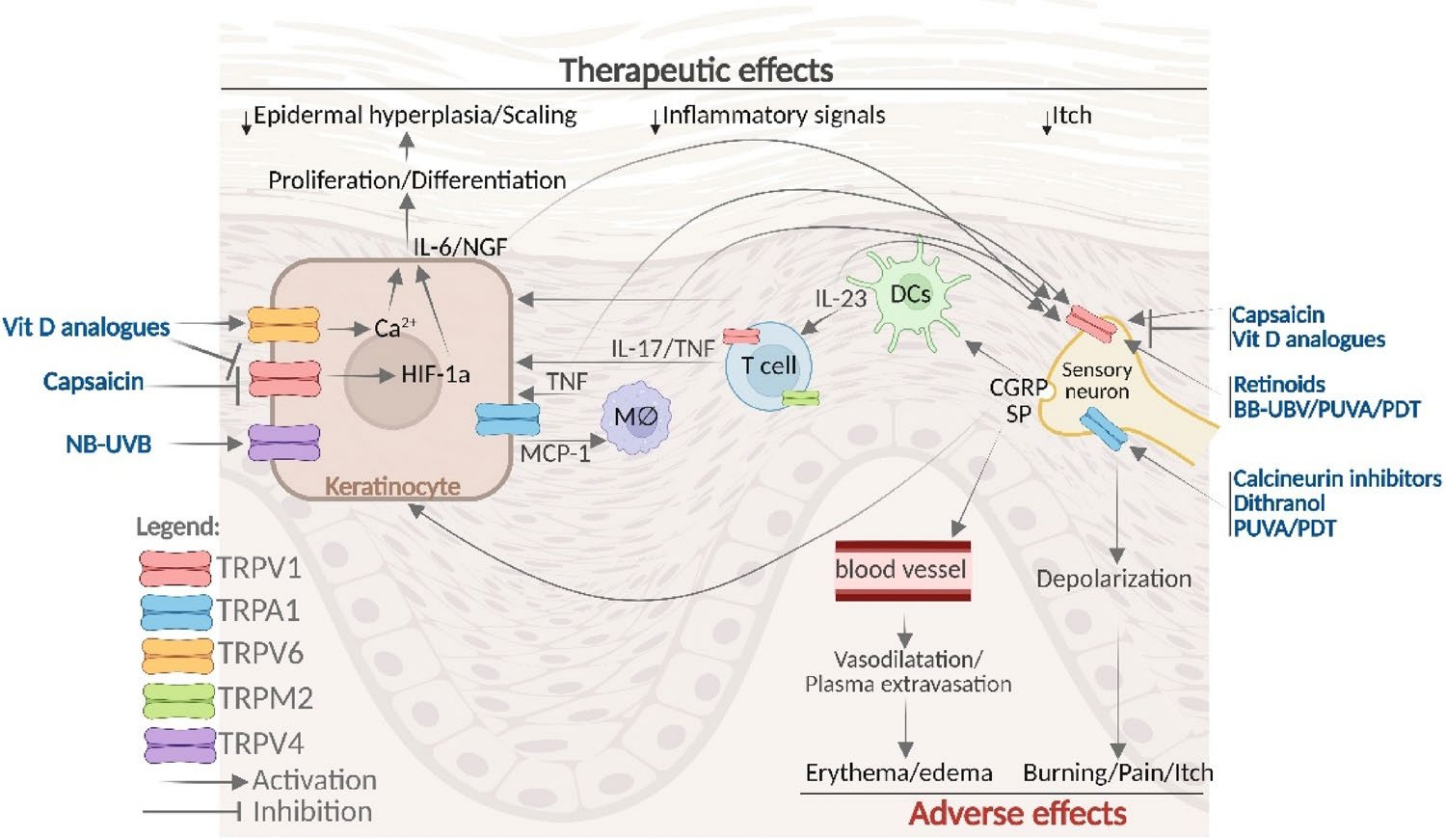
Role of TRP channels in psoriasis therapies. Several topical pharmacotherapies and phototherapies may produce adverse effects by the stimulation of TRP channels in sensory neurons, leading to early neuronal depolarization followed by itch and burning sensations, as well as to neuropeptide (SP and CGRP) release that acts in blood vessels, inducing vasodilatation and plasma extravasation followed by erythema and edema. Capsaicin, vitamin D analogs, retinoids, BB-UVB, PUVA, and PDT directly activate TRPV1 channels. In contrast, the calcineurin inhibitors, dithranol, PUVA, and PDT directly activate TRPA1 in sensory neurons, leading to adverse effects. NB-UVB-related pain is caused by the direct activation of TRPV4 receptors in keratinocytes, leading to the release of IL-6 and the stimulation of sensory neurons. Furthermore, capsaicin and vitamin D analogs may also cause late inactivation of TRPV1 (through desensitization and partial agonism, respectively), which may be related to their therapeutic effects in patients with psoriasis, including reductions in itch, inflammation, and scaling. The inactivation of the TRPV1 receptor in sensory neurons reduces not only depolarization but also CGRP release, a mediator that causes vasodilatation in blood vessels and hyperproliferation of keratinocytes. Capsaicin and vitamin D inactivation of TRPV1 also reduce the proinflammatory cytokine release by T cells and downregulate HIF-1α (an essential factor in psoriatic epidermal proliferation) in keratinocytes. The therapeutic effects of vitamin D analogs may also be related to increased TRPV6 expression, leading to calcium-dependent differentiation of keratinocytes. Anti-cytokine drugs may also exert some of their therapeutic effects by indirectly regulating TRP channels, including the downregulation of TRPV1 in sensory neurons. Anti-TNF drugs may also reduce MCP-1 release by keratinocytes via the downregulation of TRPA1 and proinflammatory cytokine release by T cells via the downregulation of TRPM2 (an effect also shared by methotrexate)

Most of the available treatments have adverse effects that limit their use. The main adverse effects of topical therapies include a burning sensation and skin irritation (Lee and Kim 2023). Other adverse effects reported include erythema and pruritus, particularly associated with phototherapy (Fritz and Salavastru 2018), as well as burning pain when using PUVA (Lee and Kim 2023). TRP channels may also represent relevant targets for current psoriasis therapies, as they are implicated in both therapeutic actions and adverse effects of some antipsoriatic drugs (Table 2 and Fig. 2).

**Table 2:** Role of TRP channels in therapeutic and adverse effects from topical therapies

Compound	Molecule	Mechanism of action	Role
Capsaicin		TRPV1 agonist	Therapeutic and adverse effects
*Vitamin D analogs*			
Calcitriol		TRPV1 partial agonist ↑mRNATRPV6	Therapeutic and adverse effects
*Calcineurin inhibitors*			
Tacrolimus		TRPA1 TRPM8 TRPV1 agonist	Adverse effects
Tazarotene		TRPV1 agonist	Adverse effects
Dithranol		TRPA1 agonist	Adverse effects

## Role of TRP channels in psoriasis therapy and its adverse effects

### Topical pharmacotherapy

#### Capsaicin

Capsaicin is the main pungent ingredient in chili peppers, selectively activates the TRPV1 receptor present in sensory neurons, which transmit information about harmful stimuli to the central nervous system, causing painful and burning sensation (Caterina et al. 1997) In addition to transmitting pain signals to the spinal cord, sensory neurons can also release peptides, such as CGRP and substance P, peripherally, producing vascular leakage and vasodilation, leading to irritation, edema and erythema at the site of application (Iyengar et al. 2017). After the initial stimulation, repeated application of low concentrations of capsaicin in the skin induces not only TRPV1 desensitization but also TRPV1-positive sensory neuron defunctionalization (facilitated by capsaicin accumulation due to its slow skin metabolism) (Chanda et al. 2008; Arora et al. 2021), which could be related to its therapeutic effect on psoriasis.

Two clinical studies investigated the potential beneficial effect of capsaicin cream in patients with psoriasis. First, a double-blind, vehicle-controlled study was performed on 44 patients to test the topical capsaicin (0.01–0.025%, administered 4–6 times daily) in the treatment of psoriasis. Three and 6 weeks after treatment initiation, they observed an improvement not only in scaling and erythema but also in overall psoriasis compared to the vehicle, with better beneficial effects observed after 6 weeks. However, adverse effects such as a burning sensation, stinging, itching, and skin redness were observed in these patients during the initial capsaicin applications, although they decreased or disappeared with continued use (Bernstein et al. 1986). Further, Ellis et al. (1993) performed a double-blind trial to evaluate the safety and efficacy of topical capsaicin (0.025%, 4 times daily, 98 patients) compared to vehicle (99 patients) in patients with pruritic psoriasis. After four and 6 weeks, capsaicin-treated patients showed a significantly greater improvement in global assessment and relief of pruritus, as well as a reduction in psoriasis severity scores, with beneficial effects again appearing after 6 weeks. As observed in the previous study, Ellis et al. (1993) also observed several adverse effects (such as burning and stinging sensations) after initial capsaicin applications, with a reduction of them after repeated treatment. Thus, the adverse effects induced by topical capsaicin treatment are related to the stimulation of TRPV1 receptors in sensory neurons. In contrast, the therapeutic effects on psoriasis are associated with the desensitization of TRPV1 receptors and the defunctionalization of TRPV1-expressing cells.

The activation of the TRPV1 receptor in sensory neurons evoked sensory neuron depolarization and release of neuropeptides (such as CGRP), resulting in burning sensation and redness of the skin, which is related to CGRP-induced vasodilatation (Sun et al. 2003). With repeated capsaicin application, TRPV1 desensitization and CGRP depletion occur (Fattori et al. 2016), which explains the attenuation of these adverse effects. On the other hand, the therapeutic response throughout treatment can also be attributed to the desensitization of TRPV1 and the subsequent dysfunction of sensory neurons that express this receptor, as CGRP released by TRPV1 activation may stimulate keratinocyte proliferation (Pepin et al. 2024).

Capsaicin may improve psoriatic lesions by desensitizing TRPV1-mediated signaling to dermal dendritic cells, thereby reducing the recruitment and activation of proinflammatory mediators. As previously cited, the defunctionalization of TRPV1-positive neurons not only reduced imiquimod-induced skin inflammation in mice but also impaired the ability of dermal dendritic cells to produce IL-23-dependent inflammatory cytokines by dermal γδ T cells and the subsequent recruitment of inflammatory cells to the skin (Riol-Blanco et al. 2014). Moreover, repeated topical application of capsaicin (0.1%) significantly reduced the epidermal hyperplasia induced by imiquimod and by IL-23 in mice as well as prevented the increase in the proinflammatory cytokines levels and the dermal infiltration of T cells, dendritic cells (and macrophages induced by imiquimod, suggesting that capsaicin disrupts the communication of TRPV1 and dermal dendritic cells, regulating the IL-23 pathway associated with reduced CGRP release (Chan et al. 2021). Finally, keratinocytes extracted from patients’ psoriatic skin exhibited hyperproliferation, which was aggravated by treatment with CGRP (Pepin et al. 2024).

In addition, TRPV1 may also be expressed and exert direct action on immune cells and keratinocytes. TRPV1 is also expressed in keratinocytes, where it modulates processes such as proliferation and differentiation (Xiao et al. 2023). Capsaicin induces calcium influx, inhibits keratinocyte proliferation, and promotes differentiation (Thio et al. 1994; Tsutsumi et al. 2011). Studies have shown that TRPV1 expression is increased in keratinocytes in psoriatic skin compared to healthy skin (Nattkemper et al. 2018). Furthermore, psoriasis patients treated with capsaicin (0.025%, three times daily for 3 weeks) downregulate hypoxia-inducible factor-1 alpha (HIF-1α), one of the most critical factors in psoriatic epidermal proliferation (Yu 2011). Apart from TRPV1 being expressed by immune cells (such as DCs, T cells, and macrophages) (Bujak et al. 2019), the direct action of capsaicin on these cells in patients with psoriasis is currently unknown. These findings demonstrate the beneficial effects of capsaicin and reinforce the neuroimmune contribution to psoriasiform inflammation, providing a nonsteroidal therapeutic alternative for the topical treatment of psoriasiform dermatitis (Chan et al. 2021).

Since TRPV1 activation and inhibition are related to the adverse and therapeutic effects of capsaicin, it seems reasonable that topical TRPV1 antagonists could be beneficial to psoriasis patients without causing limiting adverse effects. Notably, several TRPV1 antagonists are being clinically tested for the treatment of skin inflammatory diseases, exhibiting anti-inflammatory effects without significant safety concerns (Lee et al. 2018; Srour et al. 2020; Park et al. 2022).

#### Vitamin D analogs

Topical vitamin D analogs are also part of the psoriasis treatment repertoire, being a first-line therapy. Two commonly used synthetic analogs of vitamin D are calcipotriol (also known as calcipotriene), a synthetic derivative of calcitriol (1,25-dihydroxyvitamin D) (Qasem et al. 2025). The primary target of action of vitamin D analogs is the nuclear vitamin D receptor (VDR), where they act as agonists (Norman et al. 2001). Adverse effects related to use include skin irritation, characterized by burning, edema, and erythema, which typically occur on both the injured and perilesional skin and usually decrease over time (Elmets et al. 2019).

Recent studies show that vitamin D does not act exclusively through its nuclear receptor. Long et al. (2020) indicated that calcitriol is a partial agonist of TRPV1. The electrophysiology results obtained showed that vitamin D application weakly activated the TRPV1 channel, inducing Ca^2+^ influx in HEK293T cells expressing human TRPV1, with an efficacy of about 10% compared to the maximal capsaicin response. The patch-clamping technique revealed that calcitriol increases the probability and frequency of TRPV1 channel opening. As expected by a partial agonist, vitamin D decreased the Ca^2+^ influx caused by capsaicin, but not the pH-induced activity, suggesting that vitamin D interacts with TRPV1 in the same region as capsaicin. The authors confirmed this hypothesis through in silico TRPV1 structural modeling, which indeed placed vitamin D in the same binding region as capsaicin. Also, they attenuated PKC-dependent TRPV1 potentiation through interactions with a known PKC phospho-acceptor residue in TRPV1. Finally, to provide evidence for a physiological role of the interaction between vitamin D and TRPV1, the researchers employed two different cell models known to express TRPV1: mouse T cells and sensory neurons. The results obtained indicated that vitamin D reduces TRPV1-induced cytokine release from T cells and agonist-induced calcium activity in sensory neurons (Long et al. 2021; 2020). The activation of TRPV1 in sensory neurons, even if weak, may be related to some of the adverse effects produced by calcitriol, such as burning sensation, skin irritation, edema, and erythema.

On the other hand, vitamin D also inhibits hyperactive TRPV1, which could explain the therapeutic effects (including the antipruritic effect) of the vitamin in psoriasis. Indeed, it is known that vitamin D reduces the production of inflammatory cytokines by T cells (Cantorna et al. 2015); however, since VDR is not expressed in naïve T cells (Von Essen et al. 2010), this suggests that TRPV1 may mediate this mechanism. Furthermore, inhibition of TRPV1 prevents the secretion of proinflammatory cytokines, such as TNF-α and IL-17, by T cells (Majhi et al. 2015), suggesting a possible therapeutic effect of vitamin D in psoriasis through TRPV1 blockade. However, further studies are needed to confirm these assumptions.

The findings on the interaction of vitamin D and TRPV1 raise the possibility that vitamin D may also regulate other TRP members, such as TRPV6 (Long et al. 2021). Both calcium and vitamin D are known to play essential roles in keratinocyte differentiation. An altered ability to regulate intracellular Ca^2+^ influx, characterized by reduced TRPV6 expression associated with decreased vitamin D levels, may be related to an imbalance between keratinocyte proliferation and differentiation in psoriatic epidermis (Cubillos and Norgauer 2016). The silencing of TRPV6 has been shown to affect cell morphology, the development of intercellular contacts, and the ability of cells to stratify (Hoenderop et al. 2004). Calcitriol, a differentiation cofactor, increased TRPV6 mRNA and protein expression in human keratinocytes in a dose-dependent manner. This positive regulation of TRPV6 resulted in a significant increase in Ca^2+^ influx in both undifferentiated and differentiated keratinocytes. These results suggest that TRPV6 mediates, in part, the pro-differentiating effects of 1,25-dihydroxyvitamin D, increasing Ca^2+^ influx and thus promoting differentiation. They also suggest that the TRPV6 channel is a key element in the differentiation induced by Ca^2+^ or calcitriol from human keratinocytes (Lehen’kyi et al. 2007). Given the high number of undifferentiated cells in psoriasis, this suggests a potential therapeutic role for vitamin D in treating the condition. Of note, it was not investigated whether calcipotriol (the synthetic analogue of vitamin D) activates TRPV1 or regulates TRPV6. Thus, the role of TRP channels in the therapeutic or adverse effects of calcipotriol remains to be elucidated.

#### Calcineurin inhibitors

Topical calcineurin inhibitors, such as tacrolimus and pimecrolimus, are immunosuppressive drugs used in the treatment of psoriasis and atopic dermatitis (Mahajan et al. 2025), as well as having an antipruritic effect (Ständer et al. 2006). These drugs can suppress T cell activation and proliferation by binding to calcineurin and inhibiting the nuclear factor of activated T cells (NFAT) transcription factor in T cells, thereby inhibiting the synthesis of several proinflammatory cytokines that play a role in the pathogenesis of psoriasis (for a complete review of the mechanism of action, see: Noceti et al. 2014; Chen et al. 2025). Topical therapy with calcineurin inhibitors is considered effective and safe, with good tolerance, especially in sensitive areas such as the face and genital area or intertriginous psoriasis. Similar to topical capsaicin, topical calcineurin inhibitors can cause burning, itching, and erythema, which generally decrease with continued use (Reitamo et al. 2000; Abędź and Pawliczak 2019).

Some studies have indicated that the activation of TRP channels may mediate adverse effects induced by calcineurin inhibitors. Firstly, in a coculture of keratinocytes and sensory neurons isolated from pigs, tacrolimus induced the immediate release of substance P, which was prevented by the non-selective TRP channel inhibitor ruthenium red (Pereira et al. 2010). Moreover, the application of either pimecrolimus or tacrolimus to mouse skin caused the release of substance P and CGRP by sensory neurons (Ständer et al. 2007). Next, Kita et al. (2019) investigated a possible direct activation of TRP channels by tacrolimus in HEK293T cells expressing TRPA1, TRPM8, TRPV1, TRPV2, TRPV3, and TRPV4. Tacrolimus was able to activate TRPA1, and, to a lesser extent, TRPM8 or TRPV1. Since the topical application of TRPA1 activators (such as allyl isothiocyanate) causes burning, stinging, and itching sensations in human skin (Andersen et al. 2017; Joseph et al. 2021), the activation of this channel may also explain the adverse sensory effects frequently reported by patients using tacrolimus.

#### Retinoids

Retinoids are structurally related derivatives of vitamin A and are essential for various biological processes, including cell proliferation and differentiation, by downregulating the expression of proinflammatory genes (Zasada and Budzisz 2019). The commonly used systemic retinoid for severe psoriasis, especially the erythrodermic and pustular forms, is acitretin (Dogra and Yadav 2014), and tazarotene is used topically (Rendon and Barkovic 2016; Elmets et al. 2021). Topical retinoids have a favorable safety profile when compared to systemic toxicity. Local adverse effects, including erythema, itching, and burning, occur more frequently during the initial phase of treatment and are mainly associated with higher doses (Thielitz and Gollnick 2008).

The study by Yin et al. (2013) showed that synthetic and natural retinoids (including tazarotene, acitretin, and transretinoic acid) directly activate the TRPV1 channel. Retinoids evoked intracellular Ca^2+^ responses in HEK293T cells transfected with recombinant TRPV1, but not with TRPA1, TRPV3, or TRPM8. In addition, transretinoic acid was able to activate native TRPV1 expressed in dissociated mouse DRG neurons with an increase in intracellular Ca^2+^ influx, which was not observed in DRG neurons from Trpv1 knockout animals. Furthermore, transretinoic acid was able to cause the release of CGRP from the peripheral sensory nerve endings of TRPV1-positive neurons in mice. Finally, retinoids evoked pain-related behaviors in mice, which were reversed by a TRPV1 antagonist or genetic ablation. Previous studies have also shown that, in addition to direct stimulation, retinoids are also capable of increasing TRPV1 expression in neurons (EL Andaloussi-Lilja et al. 2009). Thus, TRPV1 is a receptor for retinoids and is related to the sensory side effects induced by topical retinoids.

#### Corticosteroids

Apart from TRP channels being targets for some endogenous steroids (Méndez-Reséndiz et al. 2020), there is little evidence indicating that TRP channels are targets for exogenous corticosteroids used clinically. High-potency corticosteroids (such as betamethasone and clobetasol) have been shown to have better efficacy in treating psoriasis patients than low-potency corticosteroids (such as dexamethasone and methylprednisolone) (Elmets et al. 2021). Only low-potency corticosteroids were investigated for their interaction with TRP channels. Dexamethasone indirectly activated TRPC4 in vitro by increasing the expression level of the Rasd1 protein (Wie et al. 2015). Methylprednisolone activated TRPC1 and TRPC4 by forming a complex with TRPC5 (Beckmann et al. 2017). Since TRPC1 and TRPC4 have reduced expression in psoriasis patients (see above in Role of TRP Channels in Psoriasis Pathogenesis section), the stimulation of these channels by low-potency corticosteroids does not appear to be clinically relevant in the treatment of psoriasis. However, the ability of high-potency corticosteroids to interact with TRP channels should be investigated to confirm their clinical relevance.

#### Dithranol

Dithranol (anthralin) was first synthesized in 1916 as an alternative to a natural product called chrysarobin, derived from the ararobá tree in South America, which was used to treat psoriasis. Dithranol is the most common therapeutic agent among a small number of pro-oxidants, considered effective and safe in the topical treatment of psoriasis (Lange et al. 1998). However, the exact mechanism of action of ditranol remains unknown. Even with the introduction of biologics, topical dithranol remains one of the most effective antipsoriatic agents, although it has been gradually withdrawn from current clinical practice (Benezeder et al. 2020). Dithranol demonstrated significantly better results than the placebo and similar efficacy to topical calcipotriene when used twice a day, for 1 min, after 4 weeks (Jekler and Swanbeck 1992; Grattan et al. 1997). The limitation to the use of dithranol is caused by the associated adverse effects, which include skin irritation, perilesional erythema, edema, burning, and mild to severe skin patches (Parslew and Friedmann 1999; Swinkels et al. 2003).

Our research group, for the first time, assessed the involvement of the TRPV1 in skin irritation caused by dithranol (da Silva et al. 2025). However, contrary to our hypothesis, our results suggested that the TRPV1 receptor seems to act protectively against skin irritation caused by dithranol, since TRPV1 antagonism or desensitization worsened irritation mainly in the late stage of the irritation. Although we did not observe any change in TRPV1 expression in dithranol-treated skin, TRPV1 expression in the draining lymph node was increased in dithranol-treated mice, indicating the activation of TRPV1-expressing dendritic cells during migration to the lymph nodes, thereby participating in the late stage of skin irritation caused by dithranol. Furthermore, we were unable to observe a direct interaction between dithranol and TRPV1 expressed in HEK293 cells, whereas it directly stimulated TRPA1 (R. Nassini, unpublished results). Similar to dithranol, topical TRPA1 agonists in humans cause burning, edema, and erythema (Joseph et al. 2021; Andersen et al. 2017), suggesting a potential role of TRPA1 in the adverse effects induced by dithranol.

### Systemic pharmacotherapies

Systemic non-biological (e.g., methotrexate, cyclosporine, tofacitinib, tacrolimus, and acitretin) and biological (TNF-α, IL-12/IL-23, IL-17, and IL-23 inhibitors, usually monoclonal antibodies) therapies are used by patients with moderate to severe psoriasis (≥ 10% BSA) (Menter et al. 2019, 2020). Compared to topical treatment, the role of TRP channels in the therapeutic and adverse effects of drugs used in systemic psoriasis therapy is limited and often related to the indirect action of TRP channels.

A study conducted in patients with rheumatoid arthritis (RA) indicated that neutrophils exhibit elevated oxidative stress and increased intracellular Ca^2+^ influx mediated by the TRPM2 channel, effects that were reduced by methotrexate and infliximab (a TNF-α inhibitor) treatment (Dogru et al. 2019). Notably, Özcan et al. (2021) demonstrated that TRPM2 expression is increased in blood leukocytes of patients with psoriasis, suggesting that this increase may also occur in T cells, where TRPM2 activation leads to cell proliferation and the secretion of proinflammatory cytokines (Melzer et al. 2012). However, the role of TRPM2 in the effect of systemic psoriasis therapies remains unclear.

Several cytokines involved in psoriasis pathogenesis (including TNF-α, IL-17, and IL-23) increase TRPV1 activation in sensory neurons and are capable of initiating type 17 cutaneous inflammation in the skin (Oprée and Kress 2000; Cohen et al. 2019; Luo et al. 2021). Thus, some beneficial effects of psoriasis systemic biological therapies may be indirectly related to TRPV1 downregulation in sensory neurons; however, further investigation is required to confirm this hypothesis.

In another study, Luostarinen et al. (2021) demonstrated that TNF-α incubation with cultured human HaCaT keratinocytes induced the release of monocyte chemoattractant protein-1 (MCP-1), a cytokine that is increased in patients with psoriasis but reduced when anti-TNF-α therapy is administered, serving as a local inflammatory marker to assess the severity of the disease and also the efficacy of anti-TNF-α treatment (Lembo et al. 2014). Moreover, Luostarinen et al. (2021) demonstrated that TNF-α-induced MCP-1 release was mediated by increased TRPA1 expression and activity, an effect prevented by cyclosporine and tacrolimus. In contrast, fumaric acid increased Ca^2+^ influx in cultured immune cells (splenocytes) by directly stimulating TRPA1 (Herrmann et al. 2019). Since the role of TRPA1 in psoriasis pathogenesis remains uncertain (see above in Role of TRP Channels in Psoriasis Pathogenesis section), further studies are needed to investigate the potential role of TRPA1 in systemic psoriasis therapy.

### Phototherapy

Phototherapy is a treatment option for patients who require more than topical medication or who wish to avoid systemic medicines. Commonly used ultraviolet (UV) light-based phototherapy for psoriasis includes narrowband ultraviolet B (NB-UVB, ∼311 nm), broadband ultraviolet B (BB-UVB, ∼313 nm), targeted UVB (excimer laser and excimer lamp), psoralen plus ultraviolet A (PUVA) therapy, photodynamic therapy, and others (Elmets et al. 2019). In this review, we have chosen to address the phototherapies that have been shown to correlate with TRP channels.

### NB-UVB

The initial dose for NB-UVB is based on the skin phototype or the minimum erythema dose (Dawe et al. 2011), typically administered two to three times a week (Mehta and Lim 2016). Although considered less effective than PUVA in several studies, the simplicity of treatment and cost-effectiveness favor NB-UVB as a treatment option. When compared to BB-UVB, it has also been shown to be more effective and to have fewer adverse effects (Archier et al. 2012). Among the main adverse effects are edema, pruritus, and, mainly, erythema and pain with a burning sensation (Valejo Coelho and Apetato 2016). A study by Moore et al. (2013) used a sunburn model in mice and reported that narrowband UVB (NB-UVB, ∼311 nm) activates the TRPV4 channel expressed in keratinocytes, but not TRPV3, which increases the influx of Ca^2+^ in cells and upregulates IL-6 in keratinocytes, causing them to act in an algesic manner. The use of a TRPV4 antagonist and knockout animals resulted in a reduction in pain-like sensations caused by UVB in mice, as well as less severe tissue damage and decreased macrophage and neutrophil infiltration, along with downregulation of IL-6 (Moore et al. 2013). Thus, TRPV4 seems to act painfully, being relevant in the pain caused by NB-UVB therapy, therefore being responsible for its adverse effects.

### BB-UVB

The initial dose is also based on the skin phototype or the minimum dose for erythema (Dawe et al. 2011). Few clinical studies have evaluated the safety and efficacy of this modality, and it is generally less effective than NB-UVB and PUVA (El-Mofty et al. 2010). This modality of phototherapy is older than NB-UVB and also has greater adverse effects, including erythema and pruritus (Almutawa et al. 2013). The research conducted by Cao et al. (2021) established a BB-UVB itch model in mice. This study evaluated the roles of TRPA1, TRPV1, and TRPV4 channels, demonstrating that acute exposure to BB-UVB promotes TRPV1 expression and function, which in turn induces scratching behavior in mice. The results obtained show that genetic ablation and the use of TRPV1 antagonists prevented BB-UVB-induced itching; however, TRPA1 and TRPV4 channel deficiency did not stop scratching behavior. Furthermore, TRPV1-positive DRG neurons, but not mast cells, were involved in BB-UVB-induced itching. These data suggest that the TRPV1 channel may be involved in the itch-related adverse effects induced by BB-UVB during psoriasis treatment. Therefore, inhibiting TRPV1 function may be a strategy to mitigate this adverse effect.

### PUVA

This type of phototherapy uses photosensitizing agents, known as psoralens, which sensitize target cells to the effects of UVA light (Galiatsatos et al. 2024). Psoralen is a natural furanocoumarin, and compounds derived from psoralen, such as 8-methoxypsoralen (8-MOP), 5-methoxypsoralen (5-MOP), and trimethyl psoralen, are currently used and are administered orally or topically in the form of a solution before exposure to UVA light (Menter et al. 2010). Topical PUVA is more suitable for localized psoriasis treatment. In general, they act by intercalating with DNA base pairs, forming cross-links after exposure to UVA and thus preventing DNA replication, as well as facilitating the production of reactive oxygen species that cause cell death and deplete skin lymphoid cells (Zanolli 2003; Galiatsatos et al. 2024). PUVA is considered the most effective phototherapy for plaque psoriasis (Yones et al. 2006; Archier et al. 2012); however, it also causes the most adverse effects (Almutawa et al. 2013), including erythema, itching, and burning pain (Al-Ismail et al. 2016). Studies have shown that after irradiation with UVA, psoralens undergo photosensitization reactions, generating reactive oxygen species (ROS) (Briganti et al. 2008). Furthermore, 8-MOP generates ROS regardless of illumination, contributing to both therapeutic actions and adverse effects (Yang et al. 2015).

Recently, studies have shown that TRPA1 and TRPV1 are involved in psoralen photosensitization. Human TRPA1 and TRPV1 expressed in HEK293 T cells were activated and photosensitized mainly by 8-MOP and to a lesser extent by 5-MOP. Increased Ca^2+^ influx through TRPA1 and TRPV1, respectively, was observed, and these responses were inhibited when selective channel antagonists were used. 8-MOP was also able to stimulate CGRP release in wild-type animals, but to a lesser extent in TRPV1 and TRPA1 animals. This set of data suggests that TRPA1 and TRPV1 may be primarily responsible for the adverse effects of PUVA (Babes et al. 2021).

## Photodynamic therapy

Photodynamic therapy (PDT) targets and destroys pre-malignant cells using photosensitizing chemicals, such as 5-aminolevulinic acid (ALA) and methylaminolevulinic acid (MAL) (Tandon et al. 2008). Both are precursors of protoporphyrin IX (PpIX) in the heme biosynthetic pathway. The absorption peaks of both are within the blue light range (410–420 nm), which is typical for ALA, and red (630 nm) for MAL. PpIX accumulates in psoriatic plaques, and PDT-induced apoptosis of T lymphocytes can lead to a reduction in inflammatory cytokines, improving psoriasis (Tandon et al. 2008). However, clinical studies have found minimal benefits, with low efficacy and high rates of adverse effects (Choi et al. 2015). Sensory discomfort, such as pain, is the primary adverse effect of PDT and limits its use for skin diseases (Wang et al. 2017). The perception of pain varies between patients, and it is generally more painful when performed on well-innervated areas of the skin, and the intensity of pain is associated with the size and location of the lesion. Psoriasis has high pain scores related to PDT, leading patients to drop out of treatment (Warren et al. 2009).

Babes et al. (2016) also investigated the role of the TRPA1 and TRPV1 channels in pain during exposure to PDT. Exposure to blue light activated the TRPA1 channel and TRPV1 to a lesser extent in the absence of additional photosensitization. Pretreatment with ALA or protoporphyrin IX significantly increased the light sensitivity of TRPA1 and TRPV1 through ROS generation. Genetic ablation or antagonism of these receptors resulted in a significant reduction in photodynamic therapy-induced skin CGRP release, indicating that selective TRPV1 or TRPA1 antagonists may increase pain tolerance and thus enhance the therapeutic efficiency of this therapy. On the other hand, the study of Wright et al. (2018) demonstrated that the selective antagonism of TRPV1, but not TRPA1, as well as the defunctionalization of TRPV1-positive cells, reduced the depolarization of mouse sensory neurons provoked by photodynamic therapy. In addition, menthol (a TRPM8 agonist) was able to reduce the depolarization of mouse sensory neurons and the pain-related behaviors provoked by photodynamic therapy in mice, suggesting that TRPM8 activation may have an effective analgesic action for the treatment of pain evoked by PDT (Wright et al. 2018).

## Limitations and future perspectives

There are some limitations on the studies regarding the role of TRP channels in the pathology and treatment of psoriasis. One relevant point is that several evidence for a role of TRP channels in psoriasis are based on in vitro studies, whose translatability to the clinical situation is limited. Another limitation is the reduced number of clinical studies on patients demonstrating a role of TRP channels in the effects of antipsoriatic therapy. Moreover, the direct interaction of TRP channels with drugs used by a large number of psoriasis patients (e.g. high-potency corticosteroids) has not yet been investigated. Apart from these limitations, our findings suggest that pharmacological modulation of TRP channels may offer a promising approach for developing more effective and safer therapies to treat psoriasis.

## Conclusion

Studies conducted with tissues from patients with psoriasis or using rodent models have highlighted a significant role for several TRP channels—particularly TRPV1, TRPV3, TRPV4, TRPV6, TRPC6, and TRPM8—in the pathogenesis of psoriasis. These channels are involved in key processes, including keratinocyte proliferation and differentiation, immune cell activation, and modulation of sensory neurons. In addition, the activation of TRPV1 and TRPA1 channels has been associated with the adverse effects of topical and phototherapy. Conversely, several studies have demonstrated that TRPV1 desensitization, as well as the defunctionalization of TRPV1-positive cells, can reduce psoriasis severity and pruritus in psoriasis patients.

## Data Availability

Not applicable to this article, as no new data were created or analyzed in this study.
